# Clinical application of susceptibility-weighted imaging in the evaluation of leptomeningeal collateralization

**DOI:** 10.1097/MD.0000000000013345

**Published:** 2018-12-21

**Authors:** Lijuan Yang, Song Luo

**Affiliations:** a Department of Pediatrics; b Department of Neurology, The first affiliated hospital of Bengbu Medical College, Bengbu, China.

**Keywords:** leptomeningeal collateralization, penumbra, perfusion-weighted imaging, stroke, susceptibility-weighted imaging

## Abstract

The feasibility of using susceptibility-weighted imaging (SWI) in a clinical setting was assessed for quantifying leptomeningeal collateralization.

Eighteen patients with stroke and acute infarction underwent diffusion-weighted imaging, SWI, perfusion-weighted imaging, and magnetic resonance angiography within 3 days after symptom onset. Lesions were evaluated by the Alberta Stroke Program Early CT score (ASPECTS), based on mean transit time, SWI, and cerebral blood volume (CBV).

For evaluating ischemic penumbra and leptomeningeal collateralization, the SWI-ASPECTS significantly correlated, respectively, with mean transit time and CBV-ASPECTS (Spearman test, *r* = 0.793 and 0.682; *P* < .001, both).

The SWI may be useful to quantify leptomeningeal collateralization in patients with acute cerebral infarction.

## 1 Introduction

Thrombolytic therapy with recombinant tissue plasminogen activator (rtPA) for patients with acute cerebral infarction can effectively promote revascularization and improve prognosis.^[1,2]^ Currently, thrombolytic therapy is guided by assessments of collateral circulation and the ischemic penumbra, as reflected, respectively, by cerebral blood volume (CBV) and mean transit time (MTT).^[3,4]^

Nearly one-third of patients who receive endovascular thrombolysis or stents did not achieve a satisfactory clinical prognosis,^[5]^ perhaps due to invalid recanalization.^[6,7]^ This may be associated with the presence of collateral circulation, especially the opening of leptomeningeal collaterals. Leptomeningeal collaterals provide a vascular network that may maintain enough cerebral blood to prolong or indefinitely sustain the viability of brain tissue beyond an occlusion. Good flow through collaterals has been associated with a large penumbra and small infarct core. By prolonging the survival time of the penumbra, the time window for viable reperfusion may also increase.^[6,7]^ Good collaterals therefore limit infarct core expansion and determine the final infarct volume.^[6,7]^

Early identification of leptomeningeal collaterals is crucial for the clinical treatment of patients with ischemic stroke. Some imaging techniques have limited clinical application because of the need for radionucleotides or contrast agents. Such methods include digital subtraction angiography, computed tomography (CT) angiography, and perfusion-weighted imaging (PWI).^[7]^

A relatively new high-resolution magnetic resonance imaging (MRI) technique that has been used to detect the leptomeningeal collaterals in stroke patients is susceptibility-weighted imaging (SWI).^[8]^ SWI does not require radionucleotides or contrast agents, because endovascular deoxygenated hemoglobin acts as an endogenous contrast agent. Compared with conventional imaging techniques, SWI provides good contrast-to-noise ratio for small blood vessels.^[9–11]^ In addition, numerous studies have shown that the status of the ischemic penumbra and collaterals can be obtain using SWI.^[3,9–12]^ However, there has been only one study of the feasibility of using SWI to evaluate leptomeningeal collateralization, to the best of our knowledge,^[8]^ and results were demonstrated qualitatively; leptomeningeal collateralization was not quantified.

We hypothesize that SWI can quantify leptomeningeal collaterals in stroke patients. The present study assessed the feasibility of using SWI to quantify leptomeningeal collaterals relative to more conventional imaging methods, particularly PWI. Eighteen stroke patients with acute infarction were evaluated using SWI, with comparisons to PWI, diffusion-weighted imaging (DWI), and magnetic resonance angiography (MRA).

## 2 Methods

### 2.1 Patients

The Medical Ethics Committee of Bengbu Medical College approved this study, and all patients gave informed consent prior to inclusion. The study population comprised 18 patients (15 men and 3 women; aged 53.8 ± 8.8 years, 22–65 years) with acute cerebral infarction who were admitted to the Department of Neurology at First Affiliated Hospital of Bengbu Medical College between June 2015 and June 2016.

For inclusion, all the patients were characterized by the following: new infarction locus detected by DWI; hospital admission within 3 days of symptom onset; 1st-ever ischemic stroke, or a previous stroke with hemiplegia sequelae that did not affect the neurologic score; aged 18–75 years, either gender; and a National Institutes of Health Stroke Scale (NIHSS) score of 4–20. DWI was applied as selection criteria, because higher signal intensities in DWIs can be observed in regions with acute infarction. Nevertheless, we also considered medical history, clinical symptoms, and NIHSS score. Patients with any of the following were excluded from this analysis: cerebral hemorrhage, tumor, or trauma detected by head CT; severe cardiovascular, renal, or hemorrhagic disease; history of allergy; current pregnancy; or psychologic or neurologic diseases such as autism.

For early-stage evaluation, the NIHSS score was determined on the day the patients arrived for initial neurologic evaluation. All patients underwent cranial SWI, PWI, MRI, DWI, and MRA within 3 days of symptom onset. The severity of neurologic damage was evaluated using NIHSS scores. Some patients underwent MRA 1 week after symptom onset to measure if revascularizations had occurred. None of the patients underwent any reperfusion treatments, such as thrombolytic therapy with rtPA, or endovascular interventions. Routine treatments were administered after admission, including antiplatelet aggregation drugs, aspirin, clopidogrel, or combined statin therapy.

### 2.2 Image analysis

**Figure 1:**
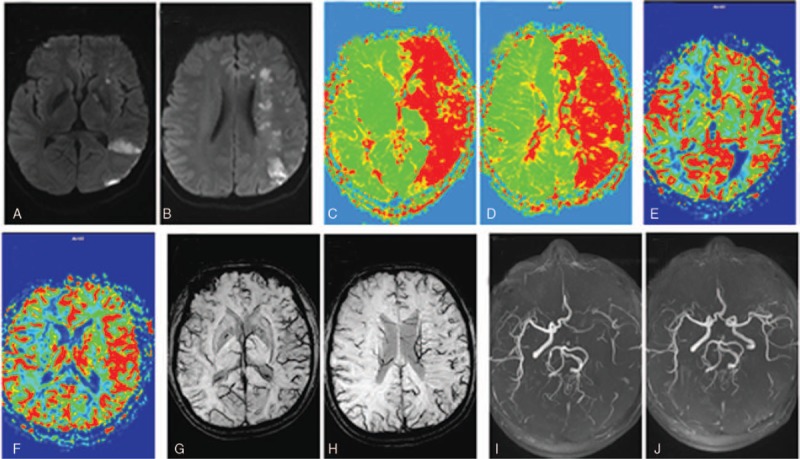
Patient experienced a cerebral infarction caused by acute occlusion of the left internal carotid artery. The admission magnetic resonance angiography (MRA) (I) and diffusion-weighted imaging (DWI) were confirmed (A and B). The patient had an National Institutes of Health Stroke scale score of 20 on admission and mean transit time (MTT) on perfusion-weighted imaging (PWI) (C and D) MTT prolonged in the area of the responsible vessel, MTT - Alberta Stroke Program Early CT score (MT-ASPECTS) was 2 points; cerebral blood volume (CBV) in the PWI showed (E and F) CBV in the lesion area increased compared with the healthy area CBV, CBV-ASPECTS was 1 point; in the susceptibility-weighted imaging (SWI) (G and H). The presence of a large number of small and prominent blood vessels (PV) in the same region, SWI-ASPECTS is 2 points; review of the head after treatment MRA (J) prompted the left internal carotid artery to achieve recanalization, mRS for 90 days after 2 points, the prognosis Better, reflected on SWI, PV grade = 2, suggesting that the cortex pia mater is better in the patient and the patient has an effective revascularization.

All images were collected as Digital Imaging and Communications in Medicine (DICOM)-format data and imported to an OsiriX image viewer for analysis. For SWI and PWI studies, the size of abnormal signal intensities or densities were evaluated in an observer-blinded fashion by 2 neuroradiologists using the 10-point quantitative topographic Alberta Stroke Program Early CT score (ASPECTS).^[13]^ For calculating ASPECTSs, 1 point was subtracted from a maximum of 10 for each area of ischemic change, including prolonged MTT, increased CBV, and prominent vessels in SWI (Fig. 1). Disagreements between the neuroradiology raters were resolved by consensus. The prominent vein on SWI was defined as a local prominence of hypointense vessels with either increased vessel number or diameter in the target area, when compared with the nontarget area^[12]^ (Fig. 1).

For late-stage neurologic evaluation, the modified Rankin scale (mRS) was used 90 days after stroke onset, with mRS ≤2 indicating a good clinical prognosis.

### 2.3 Imaging techniques

The MRI data were obtained with a 3.0T Siemens Tim Trio whole-body MRI system (Siemens, Munich, Germany) with a standard head coil. Each patient was given a conventional MRI, 3-dimensional time-of-flight MRA, spin echo-echo planar imaging (SE-EPI), magnetic resonance perfusion, and SWI. The entire cranial anatomy was scanned in the sagittal, coronal, and axial planes. The images were obtained in the following sequence: DWI; T1-weighted spin echo (T1-SE); T2-weighted fast spin echo; and T2-weighted fast fluid-attenuated inversion-recovery.

The following parameters were used for DWI: repetition time (TR), 93 milliseconds; and echo time (TE), 3800 milliseconds. For T1-SE, the parameters were: TR, 440 milliseconds; and TE, 2.5 milliseconds. For T2-weighted fast spin echo, TR, TE, and inversion time (TI) were 3000, 93, and 2371.5 milliseconds, respectively. The parameters for T2-weighted fast fluid-attenuated inversion-recovery were: slice thickness, 6 mm; interslice gap, 1.2 mm; field of view (FOV), 199 mm × 220 mm; and matrix, 464 × 512. For SWI, the magnitude and phase images were obtained with the following parameters: TR/TE, 49/40; flip angle, 15°; bandwidth, 80 kHz; slice thickness, 2 mm; 64 slices in a single slab; iPAT factor, 2; and matrix, 177 × 256 pixels.

Postprocessing was performed and 2-mm maximum intensity projection images were generated. For magnetic resonance perfusion, patients received an intravenous bolus injection of gadolinium-DTPA (diethylenetriamine penta-acetic acid; 0.2 mmol/kg) at a rate of 4.5–5.0 mL/s during the 6th SE-EPI scan, using a Medrad high-pressure syringe. Magnetic resonance perfusion was performed using the following parameters in 1 excitation: TR, 1400 milliseconds; TE, 32 milliseconds; inversion angle, 70°; FOV, 23 cm × 23 cm; slice thickness, 5 mm; interslice gap, 1.5 mm; and matrix, 128 × 128.

### 2.4 Statistical analysis

Analyses were performed using SPSS 17.0. Because the data were not normally distributed, nonparametric tests were used for comparison. The values are presented as mean and standard deviation, median and range, or number and percentage. Spearman correlation test was used to analyze the correlation between the clinical data and the SWI-, MTT-, and CBV-ASPECTS. A *P*-value <.05 was considered statistically significant.

## 3 Results

**Figure 2:**
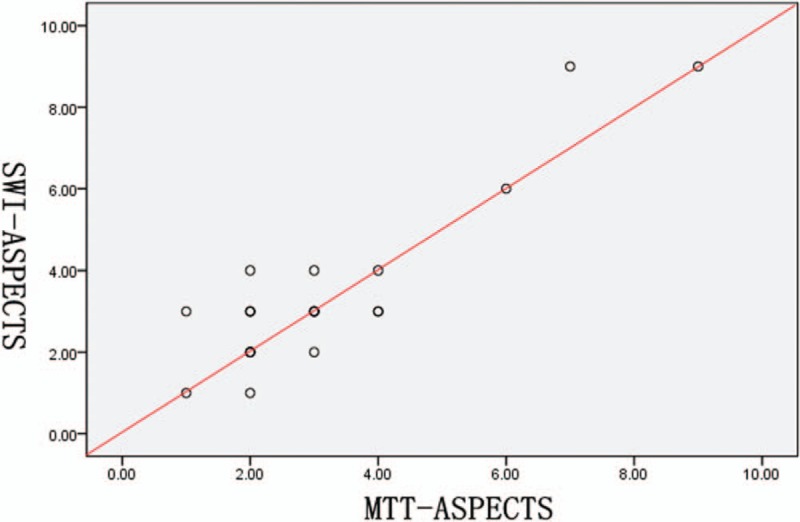
The scatter plot showing the correlation between the susceptibility-weighted imaging (SWI)- and mean transit time (MTT) - Alberta Stroke Program Early CT score (ASPECTS). The SWI-ASPECTS positively correlated with the MTT-ASPECTS (Spearman test, *r* = 0.682, *P* < .001).

**Figure 3:**
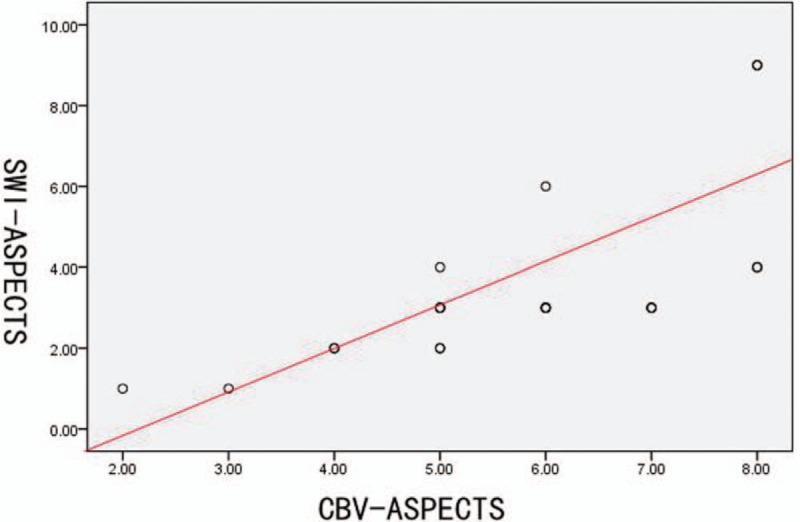
The scatter plot showing the correlation between the susceptibility-weighted imaging (SWI)- and cerebral blood volume (CBV) - Alberta Stroke Program Early CT score (ASPECTS). The SWI-ASPECTS positively correlated with the CBV-ASPECTS (Spearman test, *r* = 0.793, *P* < .001).

Eighteen patients with cerebral infarction (Table 1) were classified as to SWI cortical pial branch. SWI, MTT, and CBV were scored according to the ASPECTS criteria. There was no statistical association between the general clinical information of the patients and ASPECTS of SWI, MTT, or CBV (Table 1). There was a positive association between the ASPECTS of SWI and MTT (*r* = 0.682, *P* < .001, Fig. 2), and between the ASPECTS of SWI and CBV (*r* = 0.793, *P* < .001, Fig. 3, Table 2). These results showed that SWI can reflect collateral circulation quantitatively.

**Table 1:**
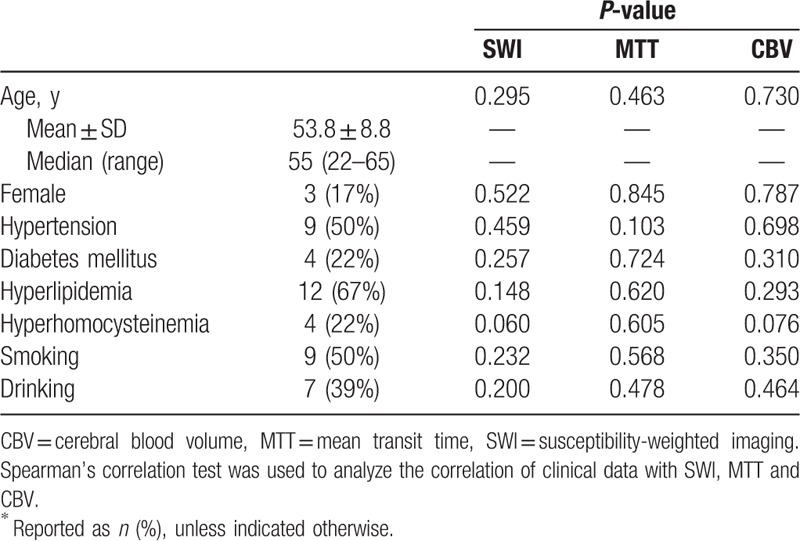
Correlations of clinical data with imaging parameters^∗^.

**Table 2:**
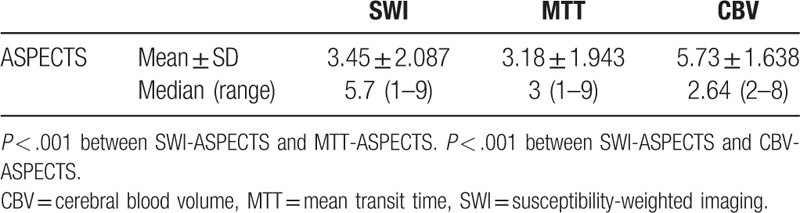
SWI-ASPECTS, CBV-ASPECTS, and MTT-ASPECTS.

In addition, 7 of the 18 patients enrolled in the review MRA had SWI cortical pial branch grade = 2. This result suggested that the cortex was soft. There were 5 improved meningeal openings and 4 revascularizations (up to 80%) were achieved.

**Figure 4:**
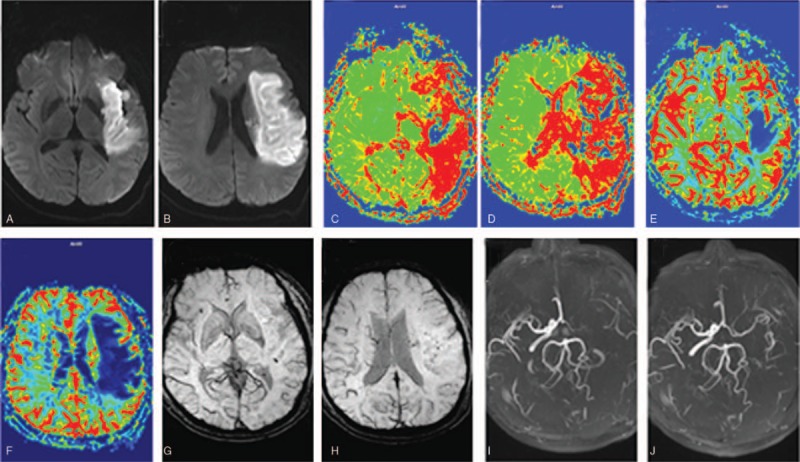
Patient showed cerebral infarction caused by an acute occlusion of the left internal carotid artery. Magnetic resonance angiography (MRA) (I) and diffusion-weighted imaging (DWI) admissions were confirmed (A and B). The National Institutes of Health Stroke scale score at admission was 16 points, and the mean transit time (MTT) at perfusion-weighted imaging (PWI) (C And D) MTT prolonged in the lesion area, MTT - Alberta Stroke Program Early CT score (ASPECTS) was 3 points; in the CWI of PWI (C and E), the cerebral blood volume (CBV) showed a smaller range of increase in CBV than in the healthy side, CBV-ASPECTS was 6 points; in the susceptibility-weighted imaging (SWI) (CWI). (G and H) In the same region, the prominent vein was absent, SWI-ASPECTS was 7 points; after the treatment, the head MRA (J) showed that the left middle cerebral artery was recanalized, and the mRS after 90 days was 4 points. The poor prognosis was reflected in the SWI. On the contrary, the prominent vein grade = 0 suggested that the cortical pia mater was not opened effectively, and although there was revascularization in the later period, the prognosis of the patient was not improved and it was an ineffective revascularization.

At the 90-day follow-up, 5 patients were better (mRS at 0–2, Fig. 1) and 2 patients had mRS = 2. Among these 7 patients, the SWIs showed cortical pial branches grade = 0, suggesting that the cortical pial circulation did not open effectively. Although the recanalization occurred later, the 90-day prognosis was poor in 2 patients (mRS was 3–4, Fig. 4).

## 4 Discussion

In this study, we hypothesized that collateral circulation had occurred, especially the cortical bronchi, which protects the ischemic penumbra. Since SWI can characterize tiny blood vessels, the feasibility of SWI was evaluated for quantifying leptomeningeal collateralization in a clinical setting. The SWI results showed that these patients had a large number of prominent vessels after routine treatment. Together with the clinical follow-up results, this indicated that revascularization occurred in some patients. These results suggest that, although patients may have missed the optimal time window for thrombolytic therapy, there was still a possibility of recanalization.

Leptomeningeal collaterals are direct arteriolo-arteriolar connections between major cerebral arteries that provide a route for retrograde filling of pial arteries distal to an occluded artery. They provide a vascular network with the potential to maintain cerebral blood flow at levels that prolong or indefinitely sustain brain tissue viability beyond an occlusion.^[14]^ Good flow through collateral pathways is associated with a large penumbra and small infarct core at baseline.^[15,16]^ By extending the survival time of penumbra, it has been shown that we can extend the time window for viable reperfusion.^[15,16]^ Healthy collaterals therefore limit infarct core expansion and determine final infarct volumes.

Collateral circulation is established during acute cerebral infarction, and has an important role in protecting the ischemic penumbra and avoiding further expansion of the core infarct size.^[16,17]^ The medial cranial circulation can be roughly divided as 1st-degree (Willis ring) or 2nd-degree (mainly referring to the ophthalmic and cortical pial branches).^[18,19]^ Liebeskind^[20]^ believed that cortical pial branches can be used as a factor to evaluate the clinical prognosis of acute cerebral infarction. In a study with mice, cortical pial branches were fully expanded within seconds of an acute occlusion of the internal carotid artery.^[21]^

Zhang et al^[22]^ analyzed the number and diameter of cortical pial blocs in the occluded middle cerebral artery blood supply area in an animal model of acute cerebral infarction to assess infarct size. Digital subtraction angiography was also performed in patients with acute cerebral infarction with acute occlusion of the middle cerebral artery, showing that retrograde perfusion of the cortical pial branch into the responsible vascular hypoperfusion region has an important role in maintaining the ischemic penumbra.^[13,18,23]^

At present, studies have suggested that CBV observed on PWI can determine the status of collateral circulation in patients with acute cerebral infarction, and can be used as a predictor of vascular recanalization after thrombolysis.^[24]^ Therefore, acute cerebral infarction can determine the establishment of collateral circulation and openness by CBV measurement.^[3,25]^ When acute cerebral infarction occurs along with low perfusion in brain tissue, the deoxygenated-to-oxygenated hemoglobin ratio varies according to the oxygen extraction fraction.^[26]^

In such a condition, there is a prominent vein in the area of the vascular blood supply on SWI, indicating the identification of cortical pia mater by SWI. When perfusion is low, the oxygen extraction fraction of the tissue increases compensatorily, and the ratio of deoxygenated-to-oxygenated hemoglobin in the blood increases, leading to an increase in the magnetic sensitivity between the tissues, which highlights many tiny blood vessels (shown as prominent veins in SWI) that are difficult to find under normal conditions. Santhosh et al^[27]^ pointed out that the prominent vein near the meninges on SWI may be collateral circulation including cortical pia mater.

Thus in this study, we speculated that the prominent veins that extended from the pia mater to the deep layers of the cortex on SWI should be the cortical pia mater. There was a significant positive correlation between the SWI- and MTT-ASPECTS, and between the SWI- and CBV-ASPECTS. In the prominent vein region near the pia mater, the CBV on PWI is increased and the MTT is prolonged compared to SWI. The underlying mechanism may be that, because of acute cerebral infarction, hypoperfusion of blood flow around the brain results in changes in the magnetic sensitivity in the lesion. Pia mater collateral circulation, which is an important part of cerebral infarction peripheral tissue collateral circulation and is formed by the slow flow of small blood vessels, showed low intravascular blood oxygen saturation. Therefore, we believe that SWI-ASPECTS can evaluate the collateral circulation in patients with acute cerebral infarction, especially the opening of cortical pial branches. The large prominent vein near the meninges on SWI can be considered the cortex pia mater.

This study also revealed the phenomena of effective and ineffective revascularization (Fig. 1), where ineffective recanalization refers to revascularization in the acute phase of cerebral infarction. Although revascularization occurs, the patient may not achieve clinical improvement.^[6,7]^ This may be due to re-occlusion of the responsible blood vessel after thrombolysis, or the collateral circulation. In particular, the degree of opening of the cortical pial branches is not perfect, and the ischemic penumbra cannot be protected before and after thrombolysis.^[6,7]^ Therefore, the clinical symptoms of patients after thrombolysis do not effectively improve. This is consistent with the results of Huang et al's^[28]^ retrospective analysis of 44 cerebral infarction patients within 2 days of onset.

The present study is limited by the small number of cases. The phenomena observed in the present study require further study for confirmation.

In this study, we believe that collateral circulation, especially in the cortical pia mater, may be an important factor in effective revascularization. The known factors affecting recanalization after thrombolysis include early thrombolysis time, blood glucose level, history of atrial fibrillation, and collateral circulation, and especially the opening of cortical pial branches.^[29–32]^ When the clinical thrombolytic treatment window in patients with acute cerebral infarction is missed, the collateral circulation may still effect revascularization, and improve the clinical prognosis. The results of the present study suggest that SWI has significant potential and applicability for assessing clinical prognosis quantitatively.

## Acknowledgments

The authors thank the Bengbu Medical College Natural Science Fund surface project (nos: BYKY1673, BYKY1790, and BYKY1688) for its support.

## Author contributions

**Conceptualization:** Song Luo.

**Investigation:** Lijuan Yang.

**Methodology:** Song Luo, Lijuan Yang.
